# Are chronic low back pain outcomes improved with co-management of concurrent depression?

**DOI:** 10.1186/1746-1340-13-8

**Published:** 2005-06-22

**Authors:** Peter Middleton, Henry Pollard

**Affiliations:** 1 Health Equilibrium, 539 Galston Road, Dural, 2158, NSW, Australia; 2 Macquarie Injury Management Group, Department Health and Chiropractic, Macquarie University, 2109, Sydney, Australia

**Keywords:** Chiropractic, depression, psychosocial, chronic low back pain.

## Abstract

**Objective:**

To discuss the role of depression in chronic lower back pain and comment on appropriate methods of screening and co-management.

**Data Sources:**

The current scientific literature was investigated using the online web databases CINAHL, Medline/PUBMED, Proquest, Meditext and from manual library searches.

**Data Extraction:**

Databases were searched from 1980 to the present (2005). Articles were searched with the key words "depression" and "low back pain". Over three hundred articles were sourced and articles were then selected on their relevance to the chronic spinal pain states that present to manual therapy practitioners.

**Data synthesis:**

Pain is a subjective awareness of peripheral nociceptive stimulation, projected from the thalamus to the cerebral cortex with each individual's pain experience being mediated by his or her psychological state. Thus a psychological component will often be associated with any painful experience. A number of studies suggest (among other things) that the incidence of depression predicts chronicity in lower back pain syndromes but that chronic lower back pain does not have the reciprocal action to predict depression.

**Conclusion:**

The aetiology of chronic pain is multifactorial. There is sufficient evidence in the literature to demonstrate a requirement to draw treatment options from many sources in order to achieve a favourable pain relief outcome. The treatment should be multimodal, including mental and emotional support, counseling and herbal advice. While a strong correlation between depression and chronic low back pain can be demonstrated, an apparent paucity of literature that specifically addresses the patient response to chiropractic treatment and concurrent psychotherapy identifies the need for prospective studies of this nature to be undertaken. It is likely that multimodal/multidisciplinary treatment approaches should be encouraged to deal with these chronic lower back pain syndromes.

## Introduction

Specific causes for acute back pain, such as infections, tumours, osteoporosis, spondyloarthropathies, and trauma actually represent a minority of pain syndromes requiring specific therapeutic approaches [1]. Chronic pain, by definition is pain "that persists for a month beyond that usual course of an acute illness or a reasonable duration for an injury to heal; that is associated with a chronic pathologic process, (and is) reocurrent at intervals for months or years"[2].

It is also important to recognise that chronic pain can occur in the absence of any pathological process (International Association for the Study of Pain {IASP} Task Force on Taxonomy 1994)[3]. The IASP describe pain as: "an unpleasant sensory and emotional experience associated with actual or potential tissue damage or described in terms of such damage". This definition reflects the view that pain is multifactorial. Engel first heralded this view in 1977 when he proposed the biopsychosocial model of pain, a model that recognises pain to involve variables such as biological, psychological, social, biomechanical dysfunction, physical deconditioning, and entrenched disability. Engel describes these variables as all being important to the generation and maintenance of chronic pain states [4].

Low back pain is defined as pain and discomfort localised below the costal margin to the inferior gluteals folds with or without leg pain as viewed from the rear [5]. Low back pain is common in western cultures with a lifetime adult population prevalence of about 70% [6], and a one-year prevalence of between 15–45%. Peak prevalence is said to occur between 35 and 55 years [7]. Much of the lower back pain is self-limiting with only 2–7% developing chronicity. Reocurrent and chronic back pain account for 75–85% of all costs associated with lower back pain [8,9]. The cause of low back pain is non-specific in most cases and serious conditions are relatively rare [10,11]. These serious conditions are usually marked by "red flag" factors that include: age of onset <20 and >50 years, recent history of violent trauma, constant progressive non mechanical pain (no relief with bed rest), thoracic pain, past medical condition of malignancy, prolonged use of corticosteroids, drug abuse, immunosuppression, HIV, systemically unwell, unexplained weight loss, widespread neurological symptoms, cauda equina syndrome, structural deformity and fever [12].

"Yellow flag" factors are those psychosocial conditions that are associated with an increased risk of developing or perpetuating chronic pain and long-term disability [13]. Yellow flag conditions include: inappropriate attitudes and beliefs about back pain (for example belief that back pain is harmful or potentially severely disabling or expectation of passive treatments rather than a belief that active participation will help), inappropriate pain behaviour (e.g. fear avoidance behaviour and reduced activity levels), work related problems or compensation issues (for example, poor work satisfaction), emotional problems (such as depression, anxiety, stress), tendency towards a low mood and withdrawal from social interaction [5].

The term acute (includes sub-acute) low back pain is defined as pain that has duration of less than three months [14]. Chronic pain is that pain which lasts for more than three months [15]. The subsequent conversion, in the absence of appropriate effective interventions, of acute back pain to chronic back pain has been found to be at times iatrogenic. This is especially so if no specific tissue can be isolated as being the cause of the pain and practitioner attempts to alleviate it prove to be only partially effectual [1]. Repeated failed treatments and various explanations of causation add to the feelings of impotence leading to catastrophising and fear avoidance behaviours (symptoms, pathology and radiological appearances are often poorly correlated) [6,16].

There often exists a strong functional overlay of psychosocial factors or yellow flags that influence this change [1]. It is recognised that there is a relationship between chronic pain and depression [17,18]. It is reported that between 50 and 65 percent of chronic pain patients also have a diagnosis for depression [19]. The treatment implications for chronic pain with the co-occurrence of depression are generally negative, with non-depressed pain patients tending to benefit from treatment more than depressed patients [20]. The relationship is complex and multifactorial, including a lower tolerance for pain in people with depression [21]. Also, an avoidance of activities that may be directly or indirectly associated with the effectiveness of the therapeutic process [22].

Evidence suggesting either a positive or negative pain outcome when psychosocial aspects of a patient's clinical presentation are addressed appears varied. This review discusses the role of depression in chronic lower back pain and comments on appropriate methods of screening and co-management.

### Data Sources

The current scientific literature was investigated using online search engines to examine the web based databases CINAHL, Medline/ PUBMED, Proquest and Meditext and from manual library searches.

### Study Period Selection

The current scientific literature was investigated from 1980 to the present (2005).

### Data Extraction

Articles were searched with the key words "depression" and "low back pain". Over three hundred articles were sourced and articles were then selected on their relevance to the chronic spinal pain states that present to manual therapy practitioners. Approximately 60 references were selected for this review.

### Data synthesis

A majority of studies suggest (among other things) that the incidence of depression predicts chronicity in pain syndromes but that chronic pain does not have the reciprocal action to predict depression.

## Results and Discussion

Pain is a subjective awareness of peripheral nociceptive stimulation, projected from the thalamus to the cerebral cortex with each individual's pain experience being mediated by his or her psychological state. Thus a psychological component will often be associated with any painful experience. But, is depression predictive of chronic low back pain? It has been suggested that the presence of depressive symptoms predicts future musculoskeletal disorders, but not vice versa [23]. Another study investigating musculoskeletal pain syndromes found that depressed patients were more likely than those without depression to report chronic pain [24]. Demonstration of psychological distress promoting low-back pain has been made [25,26]. In the study by Deyo and Diehl, a group of 1638 subjects without back pain were observed to determine the relationship between psychological distress and low-back pain. The results indicated that symptoms of psychological distress could predict the onset of new episodes of back pain. The psychological factors included depression and anxiety, which, it was stated, were involved in 16% of new episodes of low-back pain in the general population [26].

The results of chiropractic treatment of 526 patients with chronic low back pain with radiation below the knees were recorded in a prospective, longitudinal observational study [27]. The study concluded that patient outcomes were significantly better (using a Visual Analog Scale score [28]) for pain at periods of 6 and 12 months compared to those recorded in a group of 309 patients treated by medical practitioners. Depression notably, was a predictor of a poorer outcome within both groups [27].

Other literature however is equivocal on this point with some authors suggesting that addressing the issue of depression with cognitive behavioural therapy aimed at increasing patient coping strategies gives a poor prognosis towards regaining normal functional capacity [29-31].

They propose that a causal relationship exists whereby disability caused by chronic pain affecting activities of daily living leads to depressive illness. It is implied that a successful therapeutic intervention that targets low back pain could have beneficial effects on depression [32], however, such outcomes have not been conclusively demonstrated in manual therapy groups.

Despite this apparent disparity, it is worthy of note that chiropractors, as primary contact health care practitioners, should look for signs of the psychosocial aspects of chronic pain. Practitioners need to be mindful of the possibility of further exacerbating the pattern of pain by only addressing the musculoskeletal aspects of the problem [33]. Multimodal treatment approaches should be considered and implemented [34-36].

### Somatisation and its association with pain perception and depression

Somatisation is a disorder that takes the form of an expression of distress characterised by clinically significant physical symptoms that cannot be explained fully by a physical disorder. It is stated that somatisation is one of the most common of the psychiatric phenomena seen in general medical practice [37] and as such, is a presentation that chiropractors should be aware of. It is usually accompanied by degrees of depression and anxiety. These patients often hold a strong belief in the somatic symptoms they are experiencing, despite an absence of objective measures of physical disease. They can be frustrating patients for clinicians.

Patients with this somatoform disorder and a habit of 'doctor shopping' have been shown to be at a higher risk of a poor outcome after treatment for pain. This is particularly seen with quality of life issues [38,39].

BenDebba and coworkers [40] have examined the stability of the relationship established between the perception of pain and psychological distress after treatment of low back pain. Their findings suggest that the strength of the relationship between chronic pain perception and distress is related to both aspects of the patient's personality and characteristics of their illness and interestingly not to the duration of their complaint [40]. Practitioners who focus on treating somatic structures, such as chiropractors, osteopaths and physiotherapists, may tend to minimise the importance of these psychological factors in the promotion of pain [41].

### Screening for pain disability and depression

Identification of the underlying or contributing issue of depression is one that requires appropriate screening tools. Signs that may lead a practitioner to suspect that a patient requires further specific screening for an underlying psychological problem are self reported symptoms that reproduce a pain pattern inconsistent with known anatomical structures and neurology. Variable test results may similarly alert the practitioner and lead to the same conclusion.

Depression associated with low back pain and other pain populations is often different to the classical signs and symptoms of "clinical depression" [42]. Much of the emotional distress in patients with chronic pain does not include the common cognitive characteristics associated with clinical depression. These include feelings of shame, guilt and emotions of anxiety and anger. This is despite the fact that patients are often hostile toward the various medical profession(s) for not resolving their low back pain [43].

Pincus & Williams suggest that, instead of searching for a direct causal path (depression as vulnerability from developing chronic pain or chronic pain leading to development of depression) we accept that this solution does not describe the experience of most of our patients [42]. They suggest that affect and sensory information are processed in parallel, and even if one of the processing channels is more dominant the relationship is most likely to be cyclical. They conclude that we should focus on who is more vulnerable to negative affect and stress as that may allow us to help patients more effectively.

Banks and Kerns concluded in their review that "there is growing empirical evidence to suggest that depression is most commonly secondary to chronic pain" [44]. However, Pincus & Williams conclude that such a conclusion is highly compromised if the measures of depression are contaminated by somatic symptoms reflecting the effect of pain itself or its effect on illness behaviour over time. They further suggest that modern measures should attempt to integrate the disability with pain affect and relate both to psychosocial variables in order to appropriately apply a biopsychosocial model to the management of chronic low back pain conditions.

Commonly used indices and questionnaires such as the Revised Owestry Low Back Pain Disability Questionnaire (ODI)[45] and the Roland Morris Low Back Pain and Disability Questionnaire [46] are objective measures of back specific function and have high clinical utility for the recording of painful disability [47,48]. They do not however, adequately identify the possibility of psychosomatic issues in their matrices. It has been demonstrated that, of the comorbidities most adversely impacting the ODI scores, depression was ranked highly along with osteoporosis, osteoarthritis, blood disorders and headaches [49].

When investigating correlation of pain intensity measured by a Visual Analog Score, the social and anxiety/depression dimensions of the ODI do not appear to be responsive [50].

As such, the ODI should be used as a whole instrument rather than attempt to use subscale components. While the ODI has a demonstrated sensitivity and ability to measure changes in low back pain disability for the purpose of evaluating clinical progress, the lack of sensitivity to identification of possible underlying depressive states demonstrate a need to use a more appropriate instrument.

Screening with a depression specific tool such as the Beck Depression Inventory (BDI) [51] may, in those incidences of high suspicion of an underlying depressive state, be appropriate. This and other questionnaires are frequently used to identify the disability associated with the depression rather than the psychosocial factors associated with them. Therefore, care must be taken in the use of these scales. Other more recently developed questionnaires should be considered to determine such psychosocial variables. An example of such a questionnaire is the depression, anxiety, and positive outlook scale (DAPOS) [52].

The BDI is a 21 item, self-report inventory, with each item consisting of 4 statements rank-ordered in terms of increasing severity for a particular depressive symptom. In use, subjects identify the degree to which each item statement describes the way they have been feeling over the past week. Higher scores indicate greater depressive symptomatology. The BDI has been demonstrated as a sensitive measure of depression in chronic pain patients [18,53].

The fear avoidance beliefs questionnaire (FABQ) was developed to determine if patients' beliefs in physical activity and work affected their low back pain. Research suggests that specific fear-avoidance beliefs about work are strongly related to work loss due to low back pain. These findings are incorporated into a biopsychosocial model of the cognitive, affective and behavioural influences in low back pain and disability. Researchers have recommended that fear-avoidance beliefs should be considered in the management of low back pain and disability [54].

The Distress and Risk Assessment Method (DRAM) questionnaire was developed in an attempt to integrate the physical and psychological assessment of the patient. It is derived from a simple set of scales that were developed for use with low back pain patients. It can distinguish between patients with no psychological distress; those at risk of developing major psychological overlay and those that are distressed [55]. Other measures (the Spielberger Trait Anxiety Inventory, Zung Depression Scale, Modified Somatic Perception Questionnaire, and Cook-Medley Hostility Scale) have been used to predict poor outcome at surgery for lumbar surgical procedures [56].

### Treatment Options

The prevalence of major depression in patients with chronic low back pain is approximately three to four times greater than that reported in the general population [57]. Within a chiropractic patient population, Jamison demonstrated the incidence of psychological disease occurring concurrently on initial presentation to be as high as 30%. This suggests that where there is an index of suspicion evaluation of the patients' psychoemotional status needs to be considered [58].

It is argued that, in patients with clinical levels of depression, treatment modalities, including cognitive behavioural therapy and administration of anti-depressant medication, specifically targeting depressive symptomatology deserve serious consideration as an integral component of pain management programs [59].

In a multimodal treatment program of 90 patients with chronic low back pain admitted to an 8-week program of functional restoration and behavioural support the combined functional and psychological treatment resulted in significant improvements among patients by the end of the program [60]. The program consisted of 3 weeks of education, stretching and calisthenic exercises, an intensive treatment period of aerobic, functional strength and endurance exercises, education, cognitive behavioral group therapy and relaxation training in an outpatient program. The targets of psychological intervention were to alter maladaptive perceptions such as somatisation and to counteract depressive symptoms. Reduction of pain or coping with pain, were not primary targets of the program, but changing of negative perceptions and improving coping behaviour were the focus. It was found that the perception of pain was altered favourably and that coping mechanisms aimed at improving functional capacity were similarly improved [59].

A chiropractor needs to consider applying a management program with components that address the psychological aspects of the perception of chronic low back pain. All appropriate patients need to be reassured and given information that explains why they need to become active participants in their treatment. Literature designed to provide this content has been shown to assist positive outcomes in chronic lower back pain patients [60]. Collectively, these interventions may help patients become more confident and less prone to anxiety and depression. Further studies are required to determine if reassurance can alter the levels of anxiety, stress and depression in chronic low back patients.

Simple stress management advice such as yoga, relaxation techniques, an exercise regime and herbal remedies such as skullcap and valerian [61] may be a beneficial (yet unproven) adjunct to musculoskeletal treatment programs. Additionally, St. Johns Wort has been shown to beneficially effect depression [62,63], although others disagree [64]. These measures may at times have insufficient power to be of profound benefit or may only be effective in a hitherto undefined subset of patients.

While some techniques remain unproven psychological cognitive behavioural therapeutic (CBT) options have been demonstrated to have clearly advantageous outcomes with regard to decreasing the pain and distress of chronic pain syndromes [65,66].

Thus, it is likely that multiprofessional rehabilitation will evolve to provide the component parts of the management programs required to maximise outcomes in patients with chronic low back pain [34-36].

### Further research

A randomised controlled trial to examine the treatment outcomes of patients presenting for chiropractic treatment with a history of chronic back pain requires the participation of a clinical psychologist/psychiatrist employing psychometric testing. This testing would determine the presence of underlying depression and the need to administer specific treatment modalities. Groups could be broken down into control, manual therapy, manual and cognitive therapy and cognitive therapy to determine which form of therapy demonstrated the best outcomes.

Recent studies have attempted to investigate psychological outcomes in manual therapy based trials. These studies are relatively recent and provide a clear path for future research in the field [16,67,68].

## Conclusion

The aetiology of chronic pain is multifactorial. There is sufficient evidence in the literature to demonstrate a requirement to draw treatment options from many sources in order to achieve a favourable pain relief outcome. A requirement for chiropractors to adopt a broader scope of both practice and case management is suggested. Treatments administered should be multimodal with a need to include mental and emotional support, counseling and natural remedy advice (in particular St. John's Wort and possibly Valerian).

While a strong correlation between depression and chronic low back pain can be demonstrated, an apparent paucity of literature that specifically addresses the patient response to chiropractic treatment and concurrent psychotherapy identifies the need for prospective studies of this nature to be undertaken. It is likely that different modes of therapy (exercise, manipulation, mobilisation or combinations of therapy) will have different outcomes. Future studies should focus on effectiveness and the dose response characteristics of these interventions in isolation and in combination.
